# Two Approaches for a Genetic Analysis of Pompe Disease: A Literature Review of Patients with Pompe Disease and Analysis Based on Genomic Data from the General Population

**DOI:** 10.3390/children8070601

**Published:** 2021-07-16

**Authors:** Kyung-Sun Park

**Affiliations:** Department of Laboratory Medicine, Kyung Hee University School of Medicine and Kyung Hee University Medical Center, Seoul 02447, Korea; drkyungsun@gmail.com or drparkkyungsun@khu.ac.kr; Tel.: +82-2-958-8674

**Keywords:** Pompe disease, *GAA* gene, general population database, carrier frequency, genetic prevalence

## Abstract

In this study, two different approaches were applied in the analysis of the *GAA* gene. One was analyzed based on patients with Pompe disease, and the other was analyzed based on *GAA* genomic data from unaffected carriers in a general population genetic database. For this, *GAA* variants in Korean and Japanese patients reported in previous studies and in patients reported in the Pompe disease *GAA* variant database were analyzed as a model. In addition, *GAA* variants in the Korean Reference Genome Database (KRGDB), the Japanese Multi Omics Reference Panel (jMorp), and the Genome Aggregation Database (gnomAD) were analyzed. Overall, approximately 50% of the pathogenic or likely pathogenic variants (PLPVs) found in unaffected carriers were also found in real patients with Pompe disease (Koreans, 57.1%; Japanese, 46.2%). In addition, there was a moderate positive correlation (Spearman’s correlation coefficient of 0.45–0.69) between the proportion of certain PLPVs in patients and the minor allele frequency of their variants in a general population database. Based on the analysis of general population databases, the total carrier frequency for Pompe disease in Koreans and Japanese was estimated to be 1.7% and 0.7%, respectively, and the predicted genetic prevalence was 1:13,657 and 1:78,013, respectively.

## 1. Introduction

Pompe disease, or glycogen storage disease type II (MIM #232300), is a monogenic autosomal recessive disorder caused by deficiency of lysosomal alpha-glucosidase (GAA). This deficiency results in the accumulation of lysosomal glycogen in various body tissues, especially in cardiac and skeletal muscles [1,2,3]. Pompe patients who develop hypertrophic cardiomyopathy and general muscle weakness within the first year of life are classified as having classic infantile Pompe disease. Without enzyme replacement therapy (ERT), classic infantile Pompe disease is typically fatal within the first year of life. Nonclassic Pompe disease (or late-onset Pompe disease (LOPD) or childhood or adult onset Pompe disease) [3] is associated with a slowly progressive weakness of proximal muscles and respiratory dysfunction. Patients with nonclassic Pompe disease either develop symptoms without cardiac involvement before 1 year of life or develop symptoms after the first year of life. Given the benefits of early diagnosis and treatment with ERT, Pompe disease was included in the recommended uniform screening panel (the newborn screening program, NBS) in the USA [4].

In general, a research on rare diseases is conducted with the clinical and genetic information of patients. However, a huge amount of genetic information has been released to public databases, allowing us to think of new approaches to genetic diseases. Theoretically, the prevalence of a specific Mendelian disease is estimated by analyzing the proportion of unaffected carriers (carrier frequency) with the genomic information in the general population. In this study, two approaches were applied in the analysis of Pompe disease. One was based on the literature review of patients with Pompe disease reported, and the other was based on the genomic information from the general population. For these, the *GAA* gene in patients and the general population was analyzed.

## 2. Materials and Methods

### 2.1. Analysis Workflow

**Figure 1:**
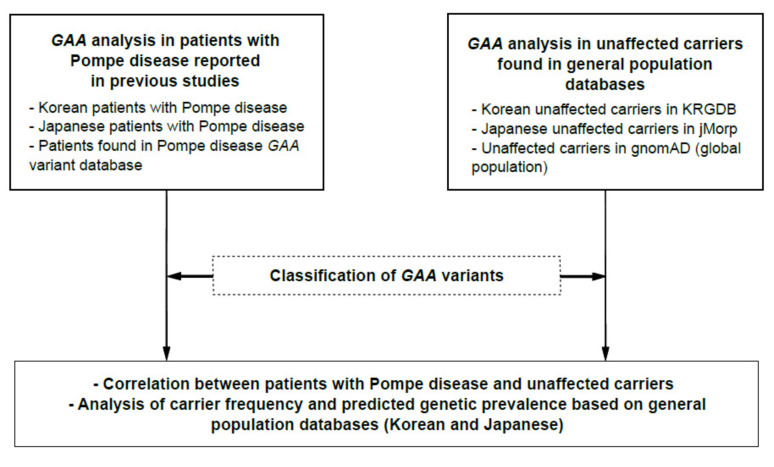
Analysis workflow in this study. The Pompe disease *GAA* variant database (http://www.pompevariantdatabase.nl/) was accessed on 16 March 2021, KRGDB (http://coda.nih.go.kr/coda/KRGDB/index.jsp) was accessed on 8 February 2021, jMorp (https://jmorp.megabank.tohoku.ac.jp/202102/variants) was accessed on 16 March 2021, and gnomAD (https://gnomad.broadinstitute.org/) was accessed on 17 March 2021.

The entire analysis workflow for the two approaches is presented in Figure 1. A literature search for Korean and Japanese patients with Pompe disease was conducted, and the causative *GAA* variants in the patients were analyzed. In this study, newborn cases without specific symptoms or signs were excluded from the analysis. For the *GAA* analysis in unaffected carriers, the *GAA* gene from both Korean and Japanese general population databases was analyzed. Recently, a database containing Korean genomic information was released, called the Korean Reference Genome Database (KRGDB, http://coda.nih.go.kr/coda/KRGDB/index.jsp, accessed on 8 February 2021), which contains 1722 Korean genomic data [5]. In the present study, *GAA* genetic variants found in KRGDB (30× coverage group, 1465 individuals) were analyzed. In addition, the Japanese Multi Omics Reference Panel (jMorp, https://jmorp.megabank.tohoku.ac.jp/202102/variants, accessed on 16 March 2021) was used to analyze *GAA* variants in the Japanese general population [6,7]. To date, the jMorp database contains the genomic data (whole-genome sequencing data) of 8380 Japanese individuals.

**Figure 2:**
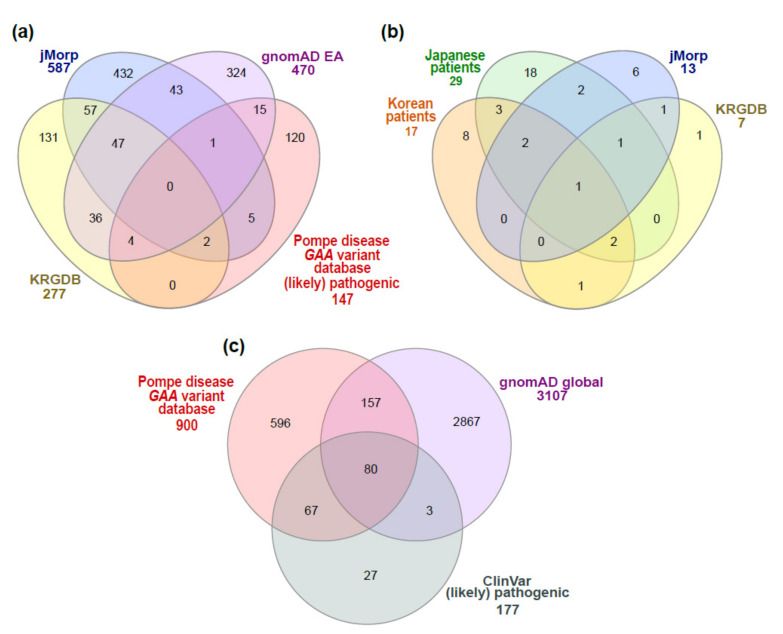
*GAA* variants found in patients with Pompe disease or in general population databases. (**a**) Number of *GAA* variants with MAF < 1% in general population databases (KRGDB, jMorp, and gnomAD) (East Asian) and number of (likely) pathogenic variants (with a review status of ≥2 gold stars in ClinVar) in the Pompe disease *GAA* variant database; (**b**) comparison of (likely) pathogenic variants found in Korean or Japanese patients, KRGDB, and jMorp; and (**c**) number of *GAA* variants in the Pompe disease *GAA* variant database, number of *GAA* variant with MAF < 1% in gnomAD (global), and number of (likely) pathogenic variants with a review status of ≥2 gold stars in ClinVar.

In order to compare *GAA* variants between general databases, excluding common variants, *GAA* variants with a minor allele frequency (MAF) < 1% in East Asians in the Genome Aggregation Database (gnomAD, https://gnomad.broadinstitute.org/, accessed on 17 March 2021, search by genomic region: chr17:78,075,380-78,093,680 (GRCh37/hg19)) [8] were compared with those in KRGDB and JMorp. For a comparison between patients with Pompe disease and the general population, *GAA* variants in Korean or Japanese patients were compared with those found in KRGDB or JMorp. In addition, *GAA* variants in the Pompe disease *GAA* variant database [9] (http://www.pompevariantdatabase.nl/, accessed on 16 March 2021) were compared with those in the general population (global) in gnomAD (https://gnomad.broadinstitute.org/, accessed on 17 March 2021). A Venn diagram for comparative analysis used InteractiVenn [10] (Figure 2). A correlation between the proportions of certain PLPVs among all PLPVs found in total patients considering the frequency of detection (for simplicity, the proportions of certain PLPVs) and the MAF of those variants in a general population database was analyzed using Spearman’s rank correlation analysis. To determine the clinical severity of Pompe disease per specific *GAA* variant, information provided by the Pompe disease *GAA* variant database was used [9] (http://www.pompevariantdatabase.nl/, accessed on 16 March 2021).

### 2.2. GAA Variant Classification

All *GAA* variants were analyzed based on NM_000152.5 (NP_000143.2) and described following the Human Genome Variation Society (HGVS) variant nomenclature standards ((http://varnomen.hgvs.org/, accessed on 17 March 2021). The *GAA* variants described in an incorrect nomenclature, which were reported in the previous literature, were not included in this study. The *GAA* variants in KRGDB, jMorp, and previous literature on Korean or Japanese patients with Pompe disease were classified or reclassified according to the 2015 American College of Medical Genetics and Genomics and the Association for Molecular Pathology standards and guidelines (2015 ACMG/AMP guidelines) [11] and specifications by a ClinGen lysosomal storage disorders expert panel (https://clinicalgenome.org/affiliation/50009/, accessed on 20 March 2021). Briefly, the PVS1, PS1, PS3, PM2, PM5, and PP4 ACMG/AMP variant criteria by the ClinGen lysosomal storage disorders expert panel (https://clinicalgenome.org/affiliation/50009/, accessed on 20 March 2021) were applied. The PM3 criterion was applied following a general recommendation by the Sequence Variant Interpretation Working Group (https://clinicalgenome.org/working-groups/sequence-variant-interpretation/, accessed on 20 March 2021); that is, each proband was given point values considering the direction of avoiding circular logic and combined values, and then the strength level for PM3 was determined. For the PP3 criterion, REVEL (>0.75 for missense variants) [12,13], MutationTaster [14], MaxEntScan (for predicted impact on splicing) [15], and spliceAI (for the predicted impact on splicing) [16] were used. For checking critical functional domains (catalytic barrel and active site) when applying the PVS1 ACMG/AMP variant criterion, Pfam (https://pfam.xfam.org/, accessed on 20 March 2021), InterPro (https://www.ebi.ac.uk/interpro/, accessed on 20 March 2021), and UniProt (https://www.uniprot.org/, accessed on 20 March 2021) were used. Among the *GAA* variants reported in the Pompe disease *GAA* variant database, variants reported as pathogenic or likely pathogenic variants (PLPVs) with a review status of ≥2 gold stars in ClinVar (https://www.ncbi.nlm.nih.gov/clinvar/, assessed on 27 April 2021) were classified as *GAA* PLPVs.

### 2.3. Analysis of Carrier Frequency and Predicted Genetic Prevalence

The carrier frequency (CF) and predicted genetic prevalence (pGP) were analyzed based on the heterozygous PLPVs. Neither the KRGDB nor the jMorp database provides information about homozygous variants. Thus (likely) pathogenic variants found in these databases were considered heterozygous variants because the general population assumes that there are no rare diseases. The CF and pGP were calculated as previously described [8,17].

## 3. Results

### 3.1. GAA Variants Found in Patients with Pompe Disease or General Population Databases

The *GAA* variants in Korean or Japanese patients with Pompe disease reported in previous studies are described in Table 1. A total of 10 studies evaluating the *GAA* variants in Korean patients with Pompe disease were reviewed [18,19,20,21,22,23,24,25,26,27]. To date, 17 different PLPVs (total of 59 PLPV alleles) in *GAA* have been reported in 32 Korean patients with Pompe disease (Table 1). *GAA* variants classified as variants of uncertain significance (VUS) because of insufficient pathogenic evidence (c.1669A>T (p.Ile557Phe) and c.2132C>G (p.Thr711Arg) reported by Kim EH et al. [27]) or *GAA* variants described in an incorrect nomenclature were excluded in this study. A total of 17 studies on Japanese patients with Pompe disease were reviewed [28,29,30,31,32,33,34,35,36,37,38,39,40,41,42,43,44], and 29 different *GAA* PLPVs (total of 130 PLPV alleles) were reported in 76 Japanese patients with Pompe disease. Of the *GAA* variants reported in 17 Japanese studies, 11 were classified as VUS and one was classified as benign, which were excluded from this analysis.

There were 277 *GAA* variants with MAF < 1% in KRGDB, 587 variants in jMorp, and 470 variants in the East Asian population in gnomAD (Figure 2a). Of those, 47 variants were included in three databases. In addition, there were 7 *GAA* PLPVs in KRGDB and 13 PLPVs in jMorp (1 suspicious PLPV (>100 bp indel) was excluded) (Table 1, Figure 2b). Most of the (likely) pathogenic variants (with a review status of ≥2 gold stars in ClinVar) reported in the Pompe disease *GAA* variant database were not found in East Asian general population databases, such as KRGDB, JMorp, and gnomAD East Asian (81.6% (120/147), Figure 2a).

Overall, a total of 46 different variants from previous Korean or Japanese studies, KRGDB, or jMorp were classified into PLPVs (Table 1). Of those, 4 PLPVs were in both Korean patients and KRGDB, 6 PLPVs were in both Japanese patients and jMorp, and only 1 PLPV (c.1857C>G, p.Ser619Arg) was found in all Korean and Japanese patients, KRGDB, and jMorp (Figure 2b). Of the 46 PLPVs, there were 21 (likely) pathogenic variants with a review status of 2 or more gold stars (2 or 3) in ClinVar (https://www.ncbi.nlm.nih.gov/clinvar/, assessed on 27 April 2021), and the other 25 were (likely) pathogenic variants with a review status of <2 gold stars (0 or 1), variants of uncertain significance, variants with conflicting interpretations of pathogenicity, or absent in ClinVar. The ACMG evidence codes for the other 25 variants are described in Table S1.

Of the 900 *GAA* variants reported in the Pompe disease *GAA* variant database, 147 variants were classified as PLPVs with a review status of ≥2 gold stars in ClinVar (https://www.ncbi.nlm.nih.gov/clinvar/, assessed on 27 April 2021) (Figure 2c). Among those, 80 PLPVs were found in gnomAD.

### 3.2. Correlation between Patients with Pompe Disease and Unaffected Carriers

**Figure 3:**
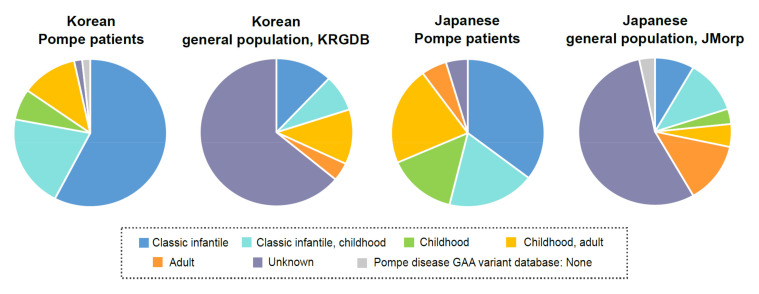
Overall distribution of clinical severity associated with *GAA* (likely) pathogenic variants detected in Korean or Japanese patients with Pompe disease and those found in unaffected carriers.

It was found that the overall distribution of clinical severity associated with *GAA* PLPVs detected in patients with Pompe disease and those in unaffected carriers differed (Figure 3). Especially, more *GAA* PLPVs associated with classic infantile Pompe disease were found in patients with Pompe disease than in unaffected carriers.

**Figure 4:**
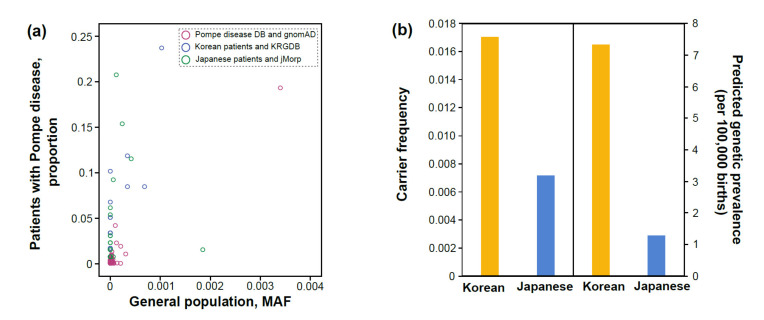
(**a**) Scatterplot of the proportion of certain (likely) pathogenic variants among all (likely) pathogenic variants found in total patients considering the frequency of detection (Y) and the minor allele frequency of those variants in a general population database (X). Purple line circles for patients found in the Pompe disease *GAA* variant database (Pompe disease DB), blue line circles for Korean patients, and green line circles for Japanese patients; (**b**) carrier frequency and predicted genetic prevalence for Pompe disease in Koreans and Japanese.

Spearman’s correlation coefficient between the proportion of certain PLPVs among all PLPVs found in total Korean patients (for simplicity, the proportion of certain PLPVs) and the MAF of their variants in KRGDB was 0.69 (*p* = 0.002), and it was 0.45 (*p* = 0.014) for Japanese patients. In addition, Spearman’s correlation coefficient between the proportion of certain PLPVs in the Pompe disease *GAA* variant database and their MAF in gnomAD (global) was 0.54 (*p* = 2.64 × 10^−12^) (Figure 4a).

### 3.3. Carrier Frequency and Predicted Genetic Prevalence Based on General Population Databases

The total CF for Pompe disease in Koreans was estimated to be 1.7%, and the pGP was 1:13,657 (7.32 per 100,000 births) based on KRGDB (Figure 4b). In addition, the CF for Pompe disease in Japanese was predicted to be 0.7%, and the pGP was 1:78,013 (1.28 per 100,000 births) based on jMorp (Figure 4b).

## 4. Discussion

The main questions in this study are how *GAA* variants detected in Pompe patients are related to those in unaffected carriers and, on the contrary, how genomic information from the healthy population reflects the likelihood of developing Pompe disease. Two aspects can be considered to analyze how much *GAA* PLPVs found in patients and unaffected carriers have in common. One is to consider the qualitative aspect and to analyze how identical the *GAA* PLPVs between two groups are. The other is the quantitative aspect, which is whether certain *GAA* PLPVs frequently found in patients with Pompe disease are also found at a high frequency in unaffected carriers. In this study, Koreans and Japanese and a wider range of ethnic groups were independently analyzed to identify questions related to Pompe disease and associated *GAA* variants.

Of the 17 different PLPVs detected in Korean patients with Pompe disease, 23.5% (4 PLPVs) were found in unaffected Korean carriers in KRGDB. In addition, 20.7% (6/29) of the PLPVs detected in Japanese patients were found in unaffected Japanese carriers in JMorp (Figure 2b and Table 1). Among the PLPVs detected in Korean or Japanese patients with Pompe disease, certain PLPVs were not found in any general population databases, such as KRGDB, jMorp, and gnomAD (e.g., c.796C>T (p.Pro266Ser), c.2171C>A (p.Ala724Asp), c.1585_1586delinsGT (p.Ser529Val), c.1696T>C (p.Ser566Pro)). This means that there are *GAA* variants that are enriched especially in patients, which contribute to the development of Pompe disease. In contrast, about 50% of the PLPVs in unaffected carriers are also found in real patients with Pompe disease (Koreans, 57.1%; Japanese, 46.2%) (Figure 2b). When considering *GAA* PLPVs found in both patients and unaffected carriers, the sum of the proportion of these PLPVs (among all PLPVs found in total patients) in patients occupied up to 50%–60% (52.5% in Korean patients and 59.2% in Japanese patients).

In addition, there was a moderate positive correlation (Spearman’s correlation coefficient of 0.45–0.69) between the proportion of certain PLPVs in patients and the MAF of their variants in a general population database in each of the three independent analyses. However, not all cases where PLPVs were detected in patients with Pompe disease are reported in the literature, so there is a limit to the accuracy of the proportion of certain PLPVs. In this study, Koreans were predicted to have higher CF and pGP than Japanese, and what is interesting is that Spearman’s correlation coefficient in Koreans (0.69) is also higher than in Japanese (0.45).

The incidence of Pompe disease has been estimated to be 1 in 40,000, but varies depending on the geographic region or population [1]. However, the incidence of Pompe disease reported by the NBS is much higher than the estimate [4,8]. Pompe disease has not yet been included in the Korean NBS program. It is important to estimate the incidence or prevalence rate of a disease when considering its inclusion in the newborn screening program. To date, the prevalence or incidence of Pompe disease in Koreans has not been studied. The pGP (1:13,657, 7.32 per 100,000 births) for Korean Pompe disease in this study is comparable to the incidence of 1:16,919 from an NBS program involving 473,738 newborn samples in Taiwan [45]. In this study, the pGP for Pompe disease in Japanese was 1:78,013 (1.28 per 100,000 births). According to a recent study of 103,204 newborns in Japan, the incidence of Pompe disease in Japanese is 1:34,401 (three patients with potential LOPD were identified) [46]. In these three newborns, [c.752C>T; c.761C>T] ([p.Ser251Leu; p.Ser254Leu]) variant was commonly detected, and additionally, c.317G>A (p.Arg106His), c.2003A>G (p.Tyr668Cys), and c.1244C>T (p.Thr415Met) were detected, respectively. According to the 2015 ACMG/AMP guidelines [11] and specifications by a ClinGen lysosomal storage disorders expert panel (https://clinicalgenome.org/affiliation/50009/, accessed on 20 March 2021), the additional three *GAA* variants are classified as VUS. Therefore, if *GAA* variants are classified according to the current guidelines and specifications, the incidence of Pompe disease in Japanese might be lower than 1:34,401.

Interestingly, there were differences in the distribution of PLPVs detected in East Asia. The c.1316T>A (p.Met439Lys) variant was the most frequently detected in Korean patients and the second most frequent in the Korean general population, but was not found in other populations in gnomAD. This variant is supposed to be a founder pathogenic variant for Korean Pompe disease. In addition, c.546G>T (p.Thr182=) was the most reported variant in Japanese patients, and none other than the Japanese general population was reported. In addition, c.1316T>A (p.Met439Lys) and c.546G>T (p.Thr182=) were only reported in Korean or Japanese patients in the Pompe disease *GAA* variant database [9] (http://www.pompevariantdatabase.nl/, last accessed on 27 April 2021). The (c.752C>T; c.761C>T) (p.Ser251Leu; p.Ser254Leu) variant has the highest AF in both KRGDB and jMorp. However, this variant was not identified in Korean patients with Pompe disease and was identified in only one Japanese patient with a homozygous status [28]. This variant has been reported as a common causative variant in Asia, but is mostly identified on the NBS (http://www.pompevariantdatabase.nl/ accessed on 27 April 2021). It is presumed that the clinical severity associated with this variant might be very mild. Therefore, Pompe disease with this variant could not be identified. Additionally, the haplotype frequency (including this variant) for developing Pompe disease might be extremely rare. The c.-32-13T>G variant is the most common pathogenic variant for European Pompe disease [1]. However, this variant was only found in KRGDB and not reported in any Korean or Japanese patients with Pompe disease. The c.2238G>C (p.Trp746Cys) variant was reported as a common pathogenic variant for Pompe disease in mainland China [2]; however, this variant has not been reported in Japanese patients.

## 5. Conclusions

In this study, two different approaches were made to study Pompe disease. One was to analyze *GAA* variants based on patients in a traditional way, and the other was to analyze how likely this disease was in the general population. To apply this analysis, the *GAA* variants found in patients and the general population were interpreted as the same criterion according to the standards/guidelines or specifications for the interpretation of genetic variants, and Pompe disease in Koreans and Japanese was analyzed as a model. In addition, *GAA* PLPVs (with a review status of ≥2 gold stars in ClinVar) in the Pompe disease *GAA* variant database and gnomAD were compared.

Although some real PLPVs may have been classified as VUS due to currently insufficient evidence and the accuracy of this analysis is limited because *GAA* variants in patients with Pompe disease have been analyzed in only those reported in previous studies, the relationship between *GAA* variants found in patients with Pompe disease and in the general population is predicted to be more than a moderate correlation.

## Figures and Tables

**Table 1:** Pathogenic or likely pathogenic variants found in Korean or Japanese patients with Pompe disease or in general population databases.

Variant	Korean Patients		Japanese Patients		General Population Databases, MAF			
	Allele Count	[Ref] (HT/HM)	Allele Count	[Ref] (HT/HM)	KRGDB ^1^	jMorp ^2^	gnomAD ^3^, East Asian	gnomAD ^3^, Global
c.-32-13T>G	0		0		0.000342	0	0.000207	0.003401
c.2T>C	0		1	[28] (1/0)	0	0	0	0
c.118C>T (p.Arg40 *)	1	[20] (1/0)	1	[28] (1/0)	0	0	0.000050	0.000014
c.169C>T (p.Gln57 *)	0		1	[28] (1/0)	0	0	0	0
c.307T>C (p.Cys103Arg)	0		0		0	0.000060	0	0
c.309C>A (p.Cys103 *)	0		2	[29] (0/1)	0	0	0	0
c.483dup (p.Lys162Glnfs*15)	0		2	[30] (2/0)	0	0	0	0
c.546G>A (p.Thr182=)	0		0		0	0.000179	0.000102	0.000030
c.546G>T (p.Thr182=)	2	[22] (1/0), [25] (1/0)	27	[28] (6/5), [34] (0/2), [35] (2/1), [36] (1/0), [37] (0/1)	0	0.000119	0	0
c.547-1G>C	0		1	[28] (1/0)	0	0	0	0
c.569G>A (p.Arg190His)	0		1	[28] (1/0)	0	0.000060	0	0.000016
c.655G>A (p.Gly219Arg)	0		1	[28] (1/0)	0	0	0	0.000018
c.670C>T (p.Arg224Trp)	0		1	[31] (1/0)	0	0	0	0.000022
(c.752C>T; c.761C>T) ((p.Ser251Leu; p.Ser254Leu)	0		2	[28] (1/0)	0.005476	0.001850	0.002759	0.000195
c.756_757insT (p.Pro253Serfs*77)	0		1	[28] (1/0)	0	0	0	0
c.796C>T (p.Pro266Ser)	1	[26] (1/0)	2	[28] (2/0)	0	0	0	0
c.841C>T (p.Arg281Trp)	0		0		0	0.000060	0	0.000205
c.875A>G (p.Tyr292Cys)	4	[18] (1/0), [19] (1/0), [21] (1/0), [23] (1/0)	0		0	0	0	0.000008
c.1156C>T (p.Gln386 *)	1	[23] (1/0)	0		0	0	0	0
c.1225dup (p.Asp409Glyfs*97)	1	[20] (1/0)	0		0	0	0	0
c.1309C>T (p.Arg437Cys)	3	[18] (1/0), [19] (1/0), [25] (1/0)	12	[28] (6/0), [33] (2/0), [38] (1/0), [39] (0/1), [40] (1/0)	0	0.000060	0	0.000008
c.1316T>A (p.Met439Lys)	14	[18] (4/0), [19] (1/0), [20] (1/0), [22] (1/0), [23] (1/0), [24] (1/0), [25] (3/0), [26] (2/0)	3	[28] (0/1), [35] (1/0)	0.001027	0	0.000384	0.000028
c.1322_1326+9del	2	[18] (1/0), [19] (1/0)	0		0	0	0	0
c.1447G>A (p.Gly483Arg)	0		0		0	0.000060	0	0.000008
c.1579_1580del (p.Arg527Glyfs*3)	2	[19] (1/0), [20] (1/0)	0		0	0	0	0.000004
c.1582_1583del (p.Gly528Leufs*2)	1	[18] (1/0)	0		0	0	0	0
c.1585_1586delinsGT (p.Ser529Val)	0		7	[32] (2/2), [41] (1/0)	0	0	0	0
c.1696T>C (p.Ser566Pro)	0		4	[28] (2/0), [30] (2/0)	0	0	0	0
c.1735G>A (p.Glu579Lys)	0		2	[28] (1/0), [42] (1/0)	0	0	0	0.000007
c.1798C>T (p.Arg600Cys)	0		20	[28] (7/0), [31] (1/0), [32] (7/1), [35] (2/0), [36] (1/0)	0	0.000239	0	0.000004
c.1822C>T (p.Arg608 *)	6	[18] (2/0), [19] (2/0), [22] (1/0), [24] (1/0)	4	[28] (1/1), [35] (1/0)	0	0	0.000051	0.000018
c.1826dup (p.Tyr609 *)	0		1	[28] (1/0)	0	0	0	0.000008
c.1857C>G (p.Ser619Arg)	7	[20] (1/0), [21] (1/0), [23] (2/0), [24] (1/0), [25] (1/0), [26] (1/0)	15	[28] (4/3), [31] (0/1), [33] (0/1), [42] (1/0)	0.000342	0.000418	0	0
c.1935C>A (p.Asp645Glu)	0		3	[32] (1/1)	0	0	0.001729	0.000124
c.1979G>A (p.Arg660His)	0		2	[31] (2/0)	0	0	0	0.000037
c.2014C>T (p.Arg672Trp)	0		0		0	0.000060	0	0.000008
c.2015G>A (p.Arg672Gln)	5	[20] (1/0), [24] (2/0), [25] (1/0), [26] (1/0)	8	[32] (2/2), [43] (0/1)	0.000343	0	0.000111	0.000021
c.2171C>A (p.Ala724Asp)	3	[18] (1/0), [19] (1/0), [25] (1/0)	0		0	0	0	0
c.2177C>G (p.Pro726Arg)	0		2	[28] (1/0), [40] (1/0)	0	0	0	0
c.2297A>G (p.Tyr766Cys)	0		1	[28] (1/0)	0	0	0	0.000025
c.2238G>C (p.Trp746Cys)	5	[19] (1/0), [22] (1/0), [24] (1/0), [25] (2/0)	0		0.000685	0	0.000351	0.000308
c.2238G>T (p.Trp746Cys)	0		0		0	0.000119	0	0
c.2326C>T (p.Gln776 *)	0		2	[33] (2/0)	0	0	0	0
c.2407_2413del (p.Gln803 *)	1	[23] (1/0)	0		0	0	0	0
c.2481+1G>A	0		1	[28] (1/0)	0	0	0	0
c.2647-7G>A	0		0		0.000342	0.000298	0	0.000018

^1^ The Korean Reference Genome Database (KRGDB, http://coda.nih.go.kr/coda/KRGDB/index.jsp, accessed on 8 February 2021). ^2^ The Japanese Multi Omics Reference Panel (jMorp, https://jmorp.megabank.tohoku.ac.jp/202102/variants, accessed on 16 March 2021). ^3^ The Genome Aggregation Database (gnomAD, https://gnomad.broadinstitute.org/, accessed on 17 March 2021). *, stop codon; Ref, references; HT, heterozygous allele count; HM, homozygous allele count; MAF, minor allele frequency.

## Data Availability

All data analyzed in this study are included in this article and its Supplementary Information files.

## Supplementary Materials

The following are available online at https://www.mdpi.com/article/10.3390/children8070601/s1: Table S1: Presumed pathogenic or likely pathogenic variants in the *GAA* gene are found in Korean or Japanese patients or in general population databases.
